# Decimated little brown bats show potential for adaptive change

**DOI:** 10.1038/s41598-020-59797-4

**Published:** 2020-02-20

**Authors:** Giorgia G. Auteri, L. Lacey Knowles

**Affiliations:** Department of Ecology and Evolutionary Biology and Museum of Zoology, University of Michigan, Ann Arbor, MI 48109 USA

**Keywords:** Conservation biology, Evolutionary ecology, Evolutionary genetics

## Abstract

The degree to which species can rapidly adapt is key to survival in the face of climatic and other anthropogenic changes. For little brown bats (*Myotis lucifugus*), whose populations have experienced declines of over 90% because of the introduced fungal pathogen that causes white-nose syndrome (WNS), survival of the species may ultimately depend upon its capacity for adaptive change. Here, we present evidence of selectively driven change (adaptation), despite dramatic nonadaptive genomic shifts (genetic drift) associated with population declines. We compared the genetic makeups of wild survivors versus non-survivors of WNS, and found significant shifts in allele frequencies of genes associated with regulating arousal from hibernation (GABARB1), breakdown of fats (cGMP-PK1), and vocalizations (FOXP2). Changes at these genes are suggestive of evolutionary adaptation, given that WNS causes bats to arouse with unusual frequency from hibernation, contributing to premature depletion of fat reserves. However, whether these putatively adaptive shifts in allele frequencies translate into sufficient increases in survival for the species to rebound in the face of WNS is unknown.

## Introduction

Events that kill large portions of populations, including naturally and anthropogenically induced disasters, increasingly threaten biodiversity^1,2^. Invasive species are a major trigger of these declines^3^, including invasive pathogens, against which native species can experience high mortality due to a lack of co-evolutionary defenses^4–6^. Introduced fungal pathogens can be particularly dangerous—they can frequently survive in the environment for extended periods, affect a relatively broad range of hosts, and can be highly virulent^7^, thereby driving mass-mortalities of native species (e.g. amphibian chytrid^8^, snake fungal disease^9^, sea fan aspergillosis^10^, and others^11–13^) as well as threatening agricultural crops^14,15^ (e.g. rice blast disease^16^ and Fusarium wilt in bananas^17^).

Although host mortalities may have little impact on fungal pathogens, the pathogens can exert incredibly strong selective pressures on their host populations^18^. A pressing conservation question is whether host populations can evolve resistance or tolerance during such epidemics—a necessary first step towards preventing extinction. Strong selective pressures might theoretically lead to an evolutionary rescue effect if host populations adapt^19^. However, acute events that kill off most members of a species also reduce the genetic diversity upon which natural selection can act, thereby limiting the capacity for adaptive change^20^.

White-nose syndrome (WNS) is a disease affecting bats, which is caused by the invasive fungus *Pseudogymnoascus destructans*^21^. This highly destructive pathogen has decimated populations of bats, with 12 North American species currently affected^22^, and some populations experiencing losses of 90–100%^23^. The fungus was first inadvertently introduced to North America by humans in 2006 (in the northeastern U.S.)^24^, and is spreading across the continent, largely via infected bats^25^. The exact mechanism of death is not known, but bats apparently die from secondary physiological complications (e.g. depleted fat reserves) associated with too frequent arousals from hibernation^26^.

Here, we conduct a genome scan to test for evidence of evolutionary changes in little brown bats (*Myotis lucifugus*) in response to WNS. The recent expansion of the fungus into our study area in 2014 combined with the staggering impact of WNS on the local population (roughly 78%)^27^ provides an opportunity to study the initial evolutionary effects of this pathogen, which continues to spread throughout the continent. Eurasian bats within the genus *Myotis*—in the native range of the pathogen—tolerate fungal growths with no noticeable mortality^28,29^. In contrast, little brown bats were the most common bats in eastern North America prior to WNS, but due to population losses, the species has now been listed as endangered by the IUCN^30^ and the federal government of Canada^31^, with a similar decision by the U.S. government pending^32^. Despite large observed declines, some individuals may have greater genetic-based tolerance or resistance to the disease, raising the potential for adaptive change in little brown bats via selective forces acting on standing genetic variation. However, dramatic population losses may confound the effectiveness of selection or purge potential adaptive variants via genetic drift. Information about these evolutionary processes can help inform the tempo and pace of management efforts for this species, by indicating which, if any, populations are adapting to the pathogen and what traits may be important for survival.

## Results

**Figure 1:**
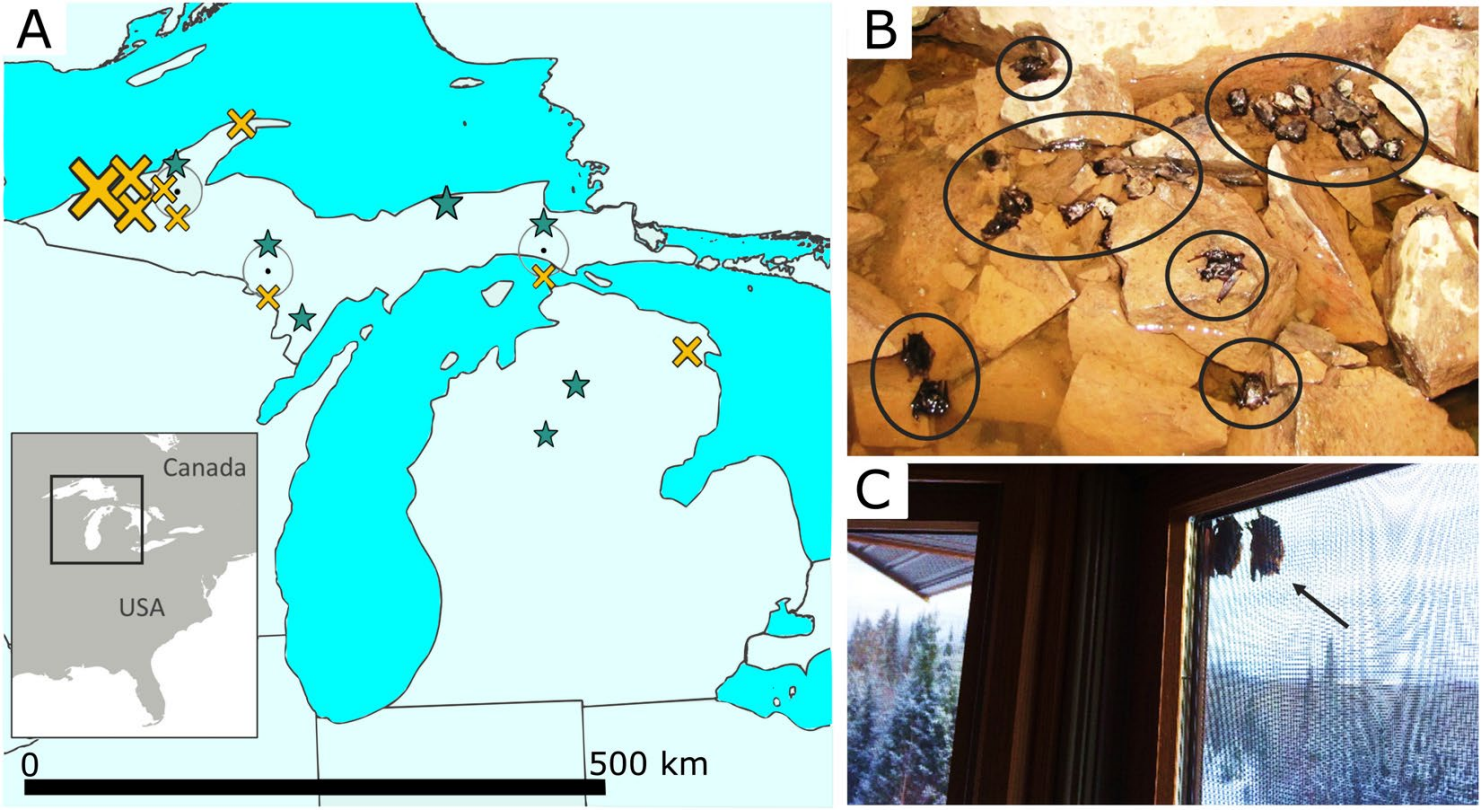
Sampling locations of little brown bats. (**A**) Sequenced survivors (*n* = 9, marked by stars) and non-survivors (*n* = 29, crosses), jittered around similar collection sites (black dots); the size of the symbol indicates relative differences in the number of samples per site (see Table S1 for details). Survivors undertake short-distance migrations away from hibernacula in spring, which is reflected in their scattered collection locations. Non-survivors are closely associated with underground hibernation sites, with most (**B**) collected within hibernacula (~26 carcasses marked by circles on the floor of a mine), although some (**C**) leave these sites prematurely, like these dead bats on the outer screen of a house <1 km from a hibernaculum (note the snowy landscape). Photo credits A. Kurta (top) and C. Rockey (bottom).

In our tests for evolutionary changes in little brown bats, we compared the genetic makeup of “survivors” and “non-survivors” of the disease (see Fig. 1) in a genome-wide survey of 19,797 single nucleotide polymorphisms (SNPs) among 14,345 loci (140 bp segments) generated from a reduced representation library (ddRadSeq^33^). We detected the effects of stochastic, non-adaptive genomic changes in otherwise neutral portions of the genome (genetic drift) reflective of the large numbers that have died from WNS in this species. Nevertheless, we also identified genetic changes (based on *F*_*ST*_-outlier analyses) that may have contributed to survival (as opposed to changes simply due to strong genetic drift), where the signature of selection can be detected by levels of genetic differentiation at a gene that exceeds background levels across the genome^34,35^. See methods for more details.

### Non-adaptive evolution associated with large number of deaths caused by WNS

**Figure 2:**
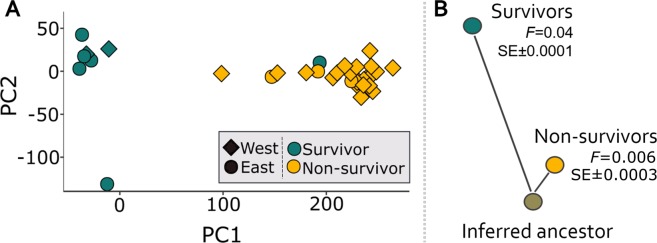
Stochastic drift induced genetic change. (**A**) PCA of survivors of WNS, with non-survivors projected onto the PC axes; PC1 explained 27% and 66% of the variance among survivors and non-survivors, respectively, and PC2 explained 13% and 6% of the variance. (**B**) The estimated degree of genetic drift (*F*, as estimated in Structure^36,37^) is an order of magnitude greater for survivors compared to non-survivors, as illustrated by the contrasting branch lengths from an inferred common ancestor.

To visualize the drift-induced changes that have occurred broadly across the genome, a PCA generated using the survivors, onto which the non-survivors were projected (Fig. 2), indicated the genomic makeup of survivors differs substantially from the non-survivors (which is robust to more stringent criteria for data filtering; Fig. S1). Quantification of the rate of evolutionary change from an inferred common ancestor showed the rate of drift is an order of magnitude higher in survivors (mean *F* = 0.04 ± SE 0.0001) relative to non-survivors (*F* = 0.006 ± 0.0003), using the *F*-model in Structure^36,37^. This amount of drift-induced genetic change (Fig. 2) is especially striking given that these changes have accumulated over, at most, three years (with most of our samples separated by just one year; Table S1), in a species that can live for well over 20 years^38,39^ and in which females typically produce one pup per year^40^.

### Selective divergence putatively driven by WNS

**Figure 3:**
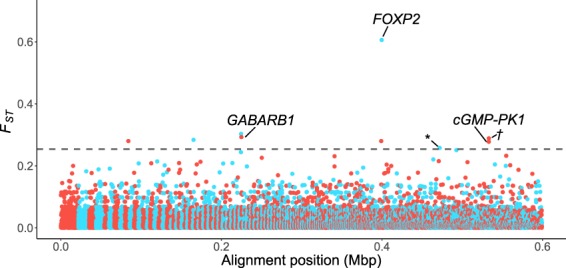
Putative loci under positive selection. AMOVA-corrected *F*_*ST*_-values of SNPs versus alignment position, highlighting the three genes that our SNPs map to, as well as an outlier SNP nearby to PLA2G7 (*), and the outlier SNP which is adjacent to CGMP-PK1 (*†*). The dashed line marks the significance threshold and alternating colors indicate different genomic scaffolds (1,214 in our dataset).

Quantification of locus-specific differentiation across the genome using *F*_*ST*_-outlier analyses identified nine SNP alleles that are significantly more common among survivors than non-survivors across all three outlier detection methods (Table S2; for details on individual genotypes see Table S3). These nine variable sites were the only outliers identified using the AMOVA-corrected *F*_*ST*_ from STACKS (Fig. 3), and were also among the outliers recognized in the two other tests (see Figs. S2 and S3). Analyses with and without four non-survivors that were collected several years prior to other samples (in 2014; Table S1) confirmed the robustness of these results to different collection dates (Figs. S2–S4).

Comparison of the nine top-candidate loci with the *M. lucifugus* reference genome (MYOLUC 2.0^41^) indicates three mRNA-coding SNPs are located in introns of annotated genes (Table S2). These three genes are: the gamma-aminobutyric acid (GABA) receptor subunit beta-1 (GABRB1; Gene ID 102432079 in the reference genome), cyclic guanosine-3′,5′-monophosphate-dependent protein kinase 1 (cGMP-PK1; Gene ID 102431010), and the forkhead box P2 protein (FOXP2; Gene ID 102423801). Two other SNPs are close to annotated genes—one was near the previously identified cGMP-PK1 gene in our dataset (3,387 bp away), and the other was near phospholipase A2 group VII (PLA2G7; Gene ID 19253; 2,747 bp away). The remaining four SNPs are relatively distant from any area of the reference genome with known function (>170,000 bp away on average).

## Discussion

We studied the genetic differences between wild little brown bats that were survivors versus non-survivors of WNS, and found evidence that there is likely a genetic component to survivorship for individuals facing this disease. This apparent adaptation has occurred very quickly since the detected evolutionary changes took place after the WNS introduction in 2014, and survivors were sampled just a few years later.

The putative selectively driven genetic changes we identify (Fig. 3) have also occurred despite dramatic nonadaptive genomic shifts (genetic drift; Fig. 2) associated with population declines due to the disease. Together, this suggests that the putative adaptive changes have resulted from very strong selective forces acting on standing genetic variation. Such rapid evolutionary changes are not unprecedented. For example, populations of the steelhead trout (*Oncorhynchus mykiss*) introduced to the central USA from coastal areas show signs of adaptation to freshwater conditions, despite small founder populations^42^. Likewise, extremely rapid phenotypic adaptation in Caribbean lizards followed a hurricane, with surviving lizards having larger toe pads which were presumably better at gripping surfaces during strong winds^43^.

The putatively adaptive SNPs among the surviving bats in our study are located within or in close proximity to four genes (cGMP-PK1, FOXP2, GABARB1, and PLA2G7), which when mapped to the annotated reference genome suggest different ways adaptive shifts might contribute to survival. GABARB1 is a receptor for the neurotransmitter GABA, which is a major neural inhibitor in the brains of vertebrates, and has also long been suspected to be involved in regulating hibernation^44^. In addition to GABA, these receptors are also sensitive to histamines^45^, which similarly help regulate hibernation in mammals^46^ and are released in response to tissue damage from WNS^47^. The importance of an individual’s sensitivity to histamines is further hinted by PLA2G7, which regulates release of histamines from mast cells^48^. Because arousals account for 80–90% of bats’ energy budget during hibernation^32^, genetic variation that contributes to even small changes in arousal frequencies could result in large differences in energy expenditures, making the difference between life and death (i.e., affecting susceptibility to WNS). We speculate that bats genetically predisposed to release fewer histamines, or be less prone to arousals induced by histamines, are better able to survive WNS through conservation of energy reserves.

Links between metabolic demands and survival are further suggested by cGMP-PK1, which was implicated by two significant SNPs in our dataset (one within the gene and one nearby). This gene is part of pathways involving cellular metabolism and breakdown of fat, and allelic variants have been linked to obesity in mammals^49,50^, which might prove beneficial for WNS-infected bats facing premature depletion of winter fat reserves. In fact, a recent study documented a post-WNS phenotypic shift towards fatter bats of this species^51^. Although this may be due to a variety of potential mechanisms, including non-evolutionary ones (see discussion in^51^), our findings suggest a genetic component to this shift.

In contrast to the SNPs linked to physiological mechanisms during winter hibernation, a SNP within FOXP2 suggests behavioral differences might confer a selective advantage. Specifically, FOXP2 is associated with vocalizations in other vertebrates, and echolocation in bats^52^. Because variation in calls is closely associated with the type of prey and habitat bats must navigate, echolocation is an important functional trait, and potentially adaptive shifts might be related to hunting proficiency, speed of developing foraging abilities in juvenile bats, or subtle differences in prey preferences. These could affect the type and amount of fat that bats store for hibernation. In addition to echolocations for hunting, bats also emit social calls. Sociality may influence the impact of the disease in this species^53^, and due to the importance of FOXP2 in communication, the gene has been linked to variations in social behavior in other species^54–56^. A more detailed study is needed to test these hypotheses, and there are possibly alternative unknown functions of FOXP2 in bats. Interestingly, no individuals in our dataset were heterozygous for this SNP.

Although outlier analyses can contain false positives, potentially inferring selectively driven differentiation when there is none^57^, we think it is unlikely the mRNA-coding SNPs we detected are statistical artifacts. The four genes we identify had putatively adaptive alleles that were entirely absent from our non-survivors (with the exception of a single allele copy in one individual). With the much greater sampling of non-survivors (*n* = 29) this difference is also not due to limited sampling (Fig. 1). An alternative consideration is that genetic drift, not selection, explains the elevated differentiation in what we identified as putatively adaptive alleles among the survivors. With inter-locus contrasts, the genome serves as the expected background for differentiation caused by drift (i.e., the expected variance in *F*_*ST*_-values in this case; Fig. 3). However demographic processes can inflate the variance of the distribution of *F*_*ST*_-values (e.g. population structure such as isolation by distance or expansion; reviewed in Hoban *et al*.^57^), potentially confounding the signals of selection and drift. Although we cannot rule out a role for non-selective processes, we note that annotation of the alleles suggests that selection is involved given that the functions are consistent with an adaptive response.

Whether the putative adaptive changes described here reflect host resistance or tolerance to the fungal pathogen has consequences for evolutionary and ecological pressures, as well as management strategies. While our study does not explicitly test whether bats survive WNS via resistance versus tolerance mechanisms, and the genomic approach we used only looked at a small portion of the genome, we found putative selection acting on non-immune genes, which suggests disease tolerance^58^ may be important. Specifically, the alleles we identify could assist some bats in “holding out” until spring, when they leave sites in which growth of the pathogen is restricted to. While infected bats do exhibit an immune response to the fungus^59^, they likely ultimately die due to secondary physiological complications linked to starvation while hibernating^26,60^. Such tolerance in little brown bats to WNS may be important for survival in both in intraspecific^61^ and interspecific^62^ contexts. However, others argue that resistance is the primary mechanism of survivorship^63^. Future work is needed to resolve this question.

## Conclusions

What the outcome of the evolutionary change we report here might be and what it bodes for the future recovery of little brown bats is not clear—it is too soon to claim that the species will be “saved” via an evolutionary rescue effect. There have been dramatic population declines, and low population sizes inherently make species vulnerable to further perturbations. Furthermore, the disease has only been present in North America for thirteen years at the time of this publication, and with little brown bats surviving to more than 20 years old in the wild^38,39^ it will take time to determine whether surviving remnant populations have sufficient reproductive and recruitment levels to avoid extinction or extirpation. However, the functions of the genes we identify suggest that for this species, and possibly other bats effected by WNS, conservation of summer foraging habitat—not just winter hibernation sites—may promote population recovery, given that the selective advantages underlying shifts in FOXP2 would most likely manifest when bats are echolocating and hunting, and not in the hibernation sites where the bats are confronted with the fungus (Fig. 1). Other genes we identified are likely subject to strong selection during winter periods of infection, but could also be important year-round (cGMP-PK1, PLA2G7, and GABRB1), given their functions in cellular metabolism. With the limited representation of the genome, there may also be selective divergence in genes not studied here. Nevertheless, even without more extensive coverage of the genome, our work hints at the multifaceted nature of selection by identifying genes whose roles differ across habitats of highly seasonal environments, and are linked to both physiological and behavioral traits.

## Materials and Methods

### Study area

We chose northern Michigan, USA, for our study because it represents a reasonably isolated population of little brown bats (Fig. 1A); WNS is present throughout our study area, and was first detected there in early 2014. We sample non-survivors from hibernation sites during the winter and survivors during the summer (when they are no longer afflicted by the pathogen). However, because the species utilizes short distance seasonal migration (typically ≤ 500 km^64^), during warmer periods they do not roost in the same sites in which they hibernate, thus the relative geographic isolation is important for assuring that bats sampled during both seasons were from the same population. Winter hibernation sites are concentrated in the northwestern portion of our study area (hibernation sites are lacking in the central and southern Michigan), and primarily consist of abandoned iron and copper mines. As a consequence, bats in our area (Fig. 1A) are isolated from other populations by two factors: the Laurentian Great Lakes and the lack of suitable subterranean hibernation sites within migration range in central and southern Michigan. The seasonal sampling of bats is necessary because WNS non-survivors can only be documented in winter areas, and disease survivors can only be identified during summer.

### Sampling of focal species

All sampled bats (Table S1) were categorized as either “survivors” or “non-survivors” of WNS. Survivors (*n* = 9) were adult bats that had been born the previous year or earlier and thus had survived at least one hibernation period with the WNS pathogen (collected during summer s of 2016–2017, see Anthony^65^ for aging methodology). Most individuals which succumb to the disease are found within the subterranean sites that afflicted species of bats rely upon in winter, and in which the fungus thrives, however some infected bats leave hibernation sites prematurely in winter in search of food or water, but quickly die due to lack of available resources and sub-freezing temperatures. Correspondingly, most non-survivors we sampled were bats found dead in or near hibernation sites during winter (collected in early 2016; *n* = 25; Fig. 1), although some tissue samples came from individuals with the pathogen that were euthanized during surveillance studies (i.e., they tested positive for the fungus; collected in early 2014; *n* = 4). Note that comparing survivors to this more general group of non-survivors makes tests for loci under selection more conservative, in that some of the euthanized bats categorized as non-survivors may not have died from WNS naturally. However, if non-survivors actually carried adaptive alleles, this would not produce a bias (i.e., make it more likely) to detect putatively selected alleles—in fact it would make such detection more difficult. In addition, all analyses were repeated excluding the euthanized bats to confirm the robustness the results.

Samples for most non-survivors (*n* = 23) were from bat carcasses found during winter either in or proximal to the caves or mines in which they were hibernating. Prior to the introduction of WNS, it was uncommon to find dead bats at hibernacula, whereas conspicuous numbers of dead individuals are found in and around these sites post-introduction of the disease (Fig. 1B), and all sites were WNS-positive at the time of collection. The accidental inclusion of bats which had died due to other causes would make it more difficult to detect adaptation in our analyses. To reduce disturbance to hibernating bats, dead bats were collected in conjunction with routine surveys by the Michigan Department of Natural Resources (MDNR) and Eastern Michigan University. Four samples were contributed by the U.S. Geological Survey National Wildlife Health Center; these bats were found during hibernation with the fungus growing on them, but were euthanized (as discussed above). Lastly, two samples of non-survivors came from the MDNR Wildlife Disease Laboratory (see details below).

Among the survivors, collection methods varied (Table S1). Three survivors were captured during summer using mist-nets, and visual inspection confirmed evidence of recovering from WNS (i.e., the presence of healing wing lesions or scars). Tissue samples were collected via small biopsy punches (2 mm diameter, one punch for each wing, Premier Medical Products Company, Plymouth Meeting, Pennsylvania, USA), after which bats were immediately released. No individual was detained for longer than 30 minutes. Eight specimens were contributed by the MDNR Wildlife Disease Laboratory, which annually receives large numbers of bats for rabies testing after they are encountered by humans or pets^66^. All individuals used in this study tested negative for the rabies virus. Six of these were considered survivors because they were submitted for testing in summer or fall; during the summer this species uses structures such as houses in addition to trees^40^ so there is no reason to believe that animals encountered by people during warmer periods were unhealthy. However, at the latitude of our study, little brown bats are not known to hibernate in buildings^40^. Consequently, any individual encountered by humans during sub-freezing periods is almost certainly on the cusp of dying from WNS. Individuals submitted to the MDNR Wildlife Disease laboratory in winter or early spring were therefore assigned to the non-survivor group (*n* = 2 in this study).

### DNA sequencing and data processing

DNA was extracted from membrane of wing tissue using DNeasy Blood and Tissue Kit (Qiagen, Valencia, CA, USA) and used to prepare a reduced representation genomic library for sequencing. Two restriction enzymes, *Eco*RI and *Mse*I, were used to digest extracted DNA (ddRadSeq^33^), to which barcodes (unique tags 10 base-pairs long) and adapters for Illumina sequencing were then ligated. Ligation and amplification were done via polymerase chain reaction (PCR), and 350 to 450 bp long fragments were size selected using Pippin Prep (Sage Science, Beverly, Massachusetts, USA). The library of 38 samples was sequenced in one HiSeq. 2500 lane (Illumina, San Diego, CA, USA), at the Centre for Applied Genomics (Toronto, Ontario, CA).

Genomic sequences were demultiplexed using the STACKS bioinformatics pipeline^67^ (v. 2.1; specifically *process rad-tags*, *gstacks*, and *populations*), and processed in conjunction with supporting programs. The first step, *process radtags*, allowed up to one mismatch in the adapter sequence and two mismatches in the barcode, with rescue of RAD-Tags allowed. A sliding window of 15% of the read length was used for an initial exclusion of any reads with a *Phred* score^68^ below 10 within the window (note additional filters of a minimum *Phred* score of 30 were applied in downstream processing, as discussed below). Of 102,419,857 initial sequences, *process radtags* removed 1,144,865 reads containing the adapter sequence, 18,775,218 reads with ambiguous barcodes, 156,274 low quality reads, and 2,495,192 reads with ambiguous RAD-Tags.

We then indexed a previously generated reference genome for the species, ftp://ftp.ncbi.nih.gov/genomes/Myotis_lucifugus (7x coverage; MYOLUC v. 2.0^41^), and mapped our sequences to the genome using the Burrows-Wheeler Alignment Program (v. 7.17) indexing and MEM algorithms, respectively^69,70^. The resulting files were filtered (-F 0x804, -q 10, -m 100), converted to .bam files, and sorted using SAMtools^71,72^ (v. 1.8-27).

The reference-based method of *gstacks* (set to remove PCR duplicates) was run using the Marukilow model^73^, minimum *Phred*^68^ score of 30, and alpha thresholds (for mean and variance) of 0.05 for discovering single nucleotide polymorphisms (SNPs). This resulted in 59,888,201 BAM records and 581,607 loci (8% of reads were excluded because they were excessively soft-clipped, and 3% had insufficient mapping qualities to be included). All remaining loci were genotyped, with a mean per-sample coverage of 10.5x ± 7.1x, a mean of 138.5 bps per locus, and consistent phasing for 88.3% of diploid loci.

*Populations* was then run with default settings and the resulting loci were filtered with a custom script in R^74^ (v. 3.5.0) to remove loci and SNPs that may be artifacts of sequencing or alignment errors (Fig. S5) based on the number of SNPs per read position, resulting in exclusion of SNPs occurring in the last 2 bp of each read. Loci with unusually high levels of diversity were also removed from consideration (threshold *θ* > 0.026), leaving 273,261 unique loci.

Using the list of vetted loci and SNPs, *populations* was then run again, retaining loci present in at least 56% of both survivors and non-survivors, ensuring a minimum sample size of at least six survivors; note the actual missing data was typically much lower (i.e., <15% in all but 7 individuals of survivors and non-survivors). This resulted in 40,963 loci (140-bp segments), of which were variable, containing 19,797 SNPs (our final SNPs), all of which had a minor allele frequency of >0.01. Minor allele thresholds of 0.01 and 0.05 were evaluated for downstream analyses, and when warranted the higher threshold was used (noted below). Mean genotyped sites per locus was 142.41 bp (*SE* ± 0.02). Because some loci contained more than one SNP, the robustness of downstream analysis to inclusion of multiple versus a single SNP per 140-bp fragment was evaluated. Main findings did not differ, thus we present analyses based on multiple SNPs per locus in the main text (see Fig. S6 for results based on a single SNP per locus).

We also checked that the data were not biased due to different levels of genetic decomposition between the survivors and non-survivors by analyzing the Guanine-Cytosine (GC) content of each sample. Specifically, raw Illumina reads (immediately after *process radtags*) of survivors were compared with the non-survivors using BBMap^75^ (v. 38.01). The proportion of GC per individual per locus was averaged across all loci for each individual using a custom script in R. Mean GC content was 43% for survivors (*n* = 9) and 42% (*n* = 29) for non-survivors, which confirmed non-survivors were not biased towards higher GC because of decomposition.

In addition, the relatedness of sampled individuals was evaluated in two ways: with *related*^76^ in R^74^ and using Plink^77^. Due to program constraints in *related*, 250 loci were randomly selected to simulate 100 pairs of individuals in each of four categories: parent-offspring, full sibling, half sibling, and unrelated. Application of the Ritland estimator of relatedness^78^ to both the simulated and empirical dataset of 1,242 filtered SNPs (see Fig. S7 caption) indicated that none of the individuals in our dataset were related with the exception of two of the non-survivors, which may be half-siblings (Fig. S7). However, the Plink^77^ analysis of 6,237 SNPs (restricted to a single SNP per locus and minor allele frequency >0.05, as per guidelines) indicated no related individuals within our dataset. We kept all individuals in downstream analyses, because the presence of a single pair of potential half siblings is not expected to influence estimates of allele frequencies or *F*_*ST*_, and removal of putatively related individuals can actually increase the error (for more details, see Waples & Anderson^79^).

Lastly, to confirm that individuals from different sampling sites within the study area could be considered one population, we used Structure^37^ (v. 2.3.4) to evaluate if genome-wide differentiation indicated a single, panmictic population. We selected the ADMIXTURE model with ‘Allele frequencies correlated’ turned on and no prior information about sampling population and explored the best supported model, considering a range of genetic clusters (i.e., *k = *1 to 5) with 10 repetitions for each *k*, for 500,000 Markov chain Monte Carlo iterations with a burn in of 50,000. Visual assessment was used to ascertain convergence by examining plots of *F*_*ST*_, alpha, and likelihood versus iterations, and to check for consistency among the ten iterations. No evidence of genetic subdivision based on geographic sampling locality was detected (see Fig. S8).

### Tests of genetic drift

Given the large numbers that have died from WNS in this species, genetic differentiation between survivors and non-survivors may result because some alleles, just by chance, will increase or decrease in frequency. These stochastic, non-adaptive genomic changes in otherwise neutral portions of the genome (genetic drift) can be particularly great when only a small proportion of the population survives, sometimes causing population bottlenecks. To visualize the drift-induced changes that occurred broadly across the genome, we conducted a principal components analysis (PCA) of the survivors, and projected the non-survivors onto the estimated PC axes, and the degree of drift was quantified using the *F*-model^36^ in STRUCTURE^37^.

The PCA was calculated for the survivors, onto which the non-survivors were projected (by applying the same scaling and centering used for survivors to the non-survivors; see Lipson *et al*.^80^). Generating a PCA in this manner is a method of visualizing differences when one group is a subset of the other (in terms of the proportion of variance), for example due to a series of founder events^80^.The PCA was performed in R^74^, in conjunction with the packages *Adegenet*^81^ (v. 2.1.1) and *Plyr*^82^ (v. 1.8.4) using the *prcomp* function. One survivor and four non-survivors were excluded from this analysis because of missing data (i.e., >50% missing loci), as were loci missing in >50% individuals (data were filtered using Plink v. 1.07^77^; see Table S1). After this, the actual missing data was <15% for all individuals except one survivor and one non-survivor, with just under 50% missing data. Missing data were then replaced with the per locus mean value across all individuals. Only genomic sites with a minor allele frequency of ≥0.05 that were variable in both survivors and non-survivors were considered, for a total of 11,462 SNPs. The PCA was repeated to confirm the robustness of the results to missing data threshold, this time using a minimum data threshold of 8.7% missing data per individual and 19% per locus (mean missing data was 1.9%), which resulted in 13,666 loci and 31 individuals being included.

We also directly estimated the amount of genetic drift between survivors and non-survivors in Structure^37^ using the *F*-model^36^ (see also Harter *et al.*^83^.). The *F*-model accounts for differences in population sizes, and has been used to quantify differences in drift between groups of contrasting sample sizes that are similar in proportion to our own^83^. For our parameter of interest, *F*, we used a prior mean and SD of 0.10, which places similar probabilities on both large and small values of *F*. To implement this Bayesian approach, we preassigned individuals to one of the two groups (survivor or non-survivor), and used a burn-in of 50,000 followed by 500,000 reps. We fixed lambda at 1, and used a uniform prior from 1 to 10 for alpha, with a standard deviation of 0.025. Three iterations were run, with different random seeds for initiating the Markov Chains.

### Tests of loci under selection

To identify genetic differences among individuals that might have contributed to their survival of WNS, we used *F*_*ST*_-outlier analyses, where the signature of selection can be detected by considering the proportional split of allelic variants between groups relative to background levels across the genome^34,35^. We identified candidate loci using three methods of outlier detection—identification of outliers via (i) the number of standard deviations from the mean using an AMOVA-corrected *F*_*ST*_^84^, (ii) by assessing confidence intervals from bootstrap permutation across loci, and (iii) measuring departure from a chi-squared distribution (detailed below). Variable sites which met all three requirements were regarded as candidate loci apparently undergoing positive selection. All tests of selection were conducted with and without the four non-survivors sampled in 2014 (collected prior to the other specimens), to confirm that the results were robust. Note that the low number of sampled survivors reflects the devastating impact of WNS on this species; despite the small sample size, it is not beyond a size in which SNPs under selection can be detected with *F*_*ST*_-outlier analyses^85^.

In our first approach, we used the AMOVA-corrected *F*_*ST*_^84^ calculated by *populations* in STACKS^35^. SNPs with an *F*_*ST*_-value of greater than nine standard deviations from the mean (mean = 0.018 ± 1 SD of 0.026) were considered outliers (similar to Willoughby *et al*.^42^). A threshold of five standard deviations is often used in detection of outlier SNPs under positive selection^42,86,87^. We increased our threshold of significance to nine standard deviations to reduce the potential for false-positives. In the second approach, confidence intervals (95% CI) were estimated using diveRsity^88^. Using the diffCalc function, Weir and Cockerham’s *F*_*ST*_^89^ was calculated for all loci, with 1,000 bootstraps performed across loci. Only loci for which the lower limit of the CI remained five SD from the mean were considered outliers. In the third approach, outliers were identified with OutFLANK^90^, which estimates the expected neutral variation of *F*_*ST*_-values under a chi-squared distribution. As per the developer guidelines^90^, we excluded loci with low expected heterozygosity (<0.1), and visually adjusted the trim functions to best fit the observed distribution (LeftTrimFraction = 0.3 and RightTrimFraction = 0.05; Fig. S9). Significance was assessed using *qvalue*^91^ in R^74^ (v. 2.12).

All results were visualized in R^74^, often in conjunction with the package *ggplot2*^92^. A custom script was used to identify SNPs which were identified as candidate loci under all three methods, and putatively selected sites were then cross-referenced with the species’ annotated reference genome^41^ to infer possible phenotypic function (see^93,94^ for additional information on the reference genome and annotation). If the SNP’s position was not within a gene, the nearest annotated areas in each direction were identified.

## Supplementary information

Supplemental Information.

## Data Availability

Genomic data (raw reads) will be made available on GenBank (SRA accession PRJNA563655). All commands (STACKS, Structure) and scripts (PCA, *F*_*ST*_) used for analyses are available on GitHub (https://github.com/giorgiaauteri/LittleBrownBats_WNSSurvivorsVsNonsurvivors).
