# Author Correction: Decimated little brown bats show potential for adaptive change

**DOI:** 10.1038/s41598-020-62444-7

**Published:** 2020-03-24

**Authors:** Giorgia G. Auteri, L. Lacey Knowles

**Affiliations:** Department of Ecology and Evolutionary Biology and Museum of Zoology, University of Michigan, Ann Arbor, MI 48109 USA

Correction to: *Scientific Reports* 10.1038/s41598-020-59797-4, published online 20 February 2020

This Article contains an error in the Data Availability section.

“Genomic data (raw reads) will be made available on GenBank (SRA accession PRJNA563655). All commands (STACKS, Structure) and scripts (PCA, FST) used for analyses are available on GitHub (https://github.com/giorgiaauteri/LittleBrownBats_WNSSurvivorsVsNonsurvivors).”

should read:

“Genomic data (raw reads) will be made available on GenBank (SRA accession PRJNA563455). All commands (STACKS, Structure) and scripts (PCA, FST) used for analyses are available on GitHub (https://github.com/giorgiaauteri/LittleBrownBats_WNSSurvivorsVsNonsurvivors).”

