# Supplementary material for: Decimated little brown bats show potential for adaptive change
Source: Sci Rep. 2020 Feb 20;10:3023. doi: 10.1038/s41598-020-59797-4 (PMC7033193; doi:10.1038/s41598-020-59797-4)
Supplement: Supplementary file 1 — Supplemental Information. [file 41598_2020_59797_MOESM1_ESM.pdf]

Supplemental Information for:

Decimated little brown bats show potential for adaptive change

Giorgia G. Auteri, L. Lacey Knowles

Table S1. Sample ID names, in conjunction with survivorship group (survivor: S; non-survivor: NS), location (generalized to county in Michigan, USA), sex (M: Male, F: Female, U:Unknown), date of collection, whether the individual was excluded from the PCA due to missing data, and the method of collection (Surveillance: bats euthanized by the federal government for WNS disease monitoring; Salvage: bats found dead in the wild apparently due to WNS; Screening: bats euthanized by the state authorities for rabies inspection; Live: bats found in the wild that were sampled and released, with band numbers “EMU YPSI” followed by 7872, 7873, and 7875, sequentially).

ID	Group	County	Sex	Date collected (dd-mm-yyyy)	Included in PCA	Collection
14NWHC02	NS	Mackinac	U	23-03-2014	Yes	Surveillance
14NWHC03	NS	Alpena	U	22-03-2014	Yes	Surveillance
14NWHC05	NS	Alpena	U	22-03-2014	Yes	Surveillance
14NWHC07	NS	Dickinson	U	26-02-2014	Yes	Surveillance
16AM01	NS	Ontonagon	F	22-02-2016	Yes	Salvage
16AM02	NS	Ontonagon	M	22-02-2016	Yes	Salvage
16AM03	NS	Ontonagon	M	22-02-2016	No	Salvage
16AM04	NS	Ontonagon	M	22-02-2016	Yes	Salvage
16AM05	NS	Ontonagon	F	22-02-2016	Yes	Salvage
16CM01	NS	Ontonagon	M	20-02-2016	No	Salvage
16CM03	NS	Ontonagon	F	20-02-2016	No	Salvage
16CM04	NS	Ontonagon	F	20-02-2016	Yes	Salvage
16CM05	NS	Ontonagon	M	20-02-2016	Yes	Salvage
16CM06	NS	Ontonagon	M	20-02-2016	Yes	Salvage
16CM07	NS	Ontonagon	F	20-02-2016	No	Salvage
16CM08	NS	Ontonagon	M	20-02-2016	Yes	Salvage
16CM09	NS	Ontonagon	M	20-02-2016	Yes	Salvage
16CM10	NS	Ontonagon	M	20-02-2016	Yes	Salvage
16CM11	NS	Ontonagon	F	20-02-2016	Yes	Salvage
16CM12	NS	Ontonagon	F	20-02-2016	Yes	Salvage
16DM01	NS	Keweenaw	M	21-02-2016	Yes	Salvage
16DM02	NS	Keweenaw	F	21-02-2016	Yes	Salvage
16FS01	NS	Ontonagon	F	22-02-2016	Yes	Salvage
16FS02	NS	Ontonagon	M	22-02-2016	Yes	Salvage
16FS03	NS	Ontonagon	M	22-02-2016	Yes	Salvage
16FS04	NS	Ontonagon	M	22-02-2016	Yes	Salvage
16FS05	NS	Ontonagon	U	22-02-2016	Yes	Salvage
16RL01	NS	Gogebic	U	01-01-2016	Yes	Screening

16RL03	NS	Houghton	U	03-03-2016	Yes	Screening
16RL05	S	Houghton	M	12-04-2016	Yes	Screening
16RL06	S	Dickinson	M	04-04-2016	No	Screening
16RL07	S	Missaukee	F	18-04-2016	Yes	Screening
16RL08	S	Crawford	M	26-07-2016	Yes	Screening
16RL09	S	Mackinac	F	17-08-2016	Yes	Screening
16RL10	S	Menominee	F	20-09-2016	Yes	Screening
17PR01	S	Alger	F	17-07-2017	Yes	Live
17PR03	S	Alger	F	17-07-2017	Yes	Live
17PR04	S	Alger	F	17-07-2017	Yes	Live

Table S2. Information on the nine significantly differentiated SNPs with respect to the reference genome. The scaffold and position of the SNP can be used to identify the location in the reference genome (ftp://ftp.ncbi.nih.gov/genomes/Myotis_lucifugus/Gnomon/). F_{ST} is the AMOVA-corrected F_{ST} [84] as calculated in STACKS[67]. For SNPs not located within mRNA sequences, we located the nearest known annotated areas (upstream or downstream) in the scaffold.

**Outlier**	**Scaffold**	**Position**	F_{ST}	**Gene**	**Distance (bp) & region**	**Function**
1	NW_005871056.1	13633675	0.2800	—	52,169 upstream of exon	Unknown (similar to multiple proteins)
2	NW_005871075.1	3019927	0.2840	—	18,912 upstream of exon	Uncharacterized
3	NW_005871095.1	6433232	0.3033	—	116,727 upstream of exon	Unknown (similar to multiple proteins)
4	NW_005871096.1	98257	0.2929	GABRB1	Within mRNA intron	Regulates arousal from hibernation
5	NW_005871218.1	2491166	0.2800	—	25,913 downstream of exon	Uncharacterized
6	NW_005871219.1	1301763	0.6061	FOXP2	Within mRNA intron	Vocalizations, echolocation
7	NW_005871329.1	378057	0.2579	*PLA2G7**	2,747 upstream of PLA2G7 exon	Regulates release of histamine from mast cells
8	NW_005871536.1	273112	0.2778	cGMP-PK1	Within mRNA intron	Regulates breakdown of fats
9	NW_005871536.1	339572	0.28875	*cGMP-PK1**	3,387 upstream of cGMP-PK1 coding sequence	See above

*These SNPs do not fall within the listed genes, but adjacent to them.

Table S3. Genotypes for each individual for the nine candidate SNPs. Major alleles were associated with non-survivors (the majority of our samples). Genotypes homozygous for minor alleles are shaded dark green, and heterozygotes are light green.

	Outliers								
Sample ID	1	2	3	4	5	6	7	8	9
mortality_14NWHC02	CC	AA	TT	GG	GG	GG	CC	CC	TT
mortality_14NWHC03	—	—	TA	GG	GG	GG	CC	CC	TT
mortality_14NWHC05	CC	AA	TT	GG	GG	GG	CC	CC	TT
mortality_14NWHC07	—	AA	TT	GG	GG	GG	CC	CC	TT
mortality_16AM01	CC	AA	—	GG	GG	GG	CC	CC	—
mortality_16AM02	CC	AA	TT	GG	GG	GG	CC	CC	TT
mortality_16AM03	—	—	—	GG	—	—	CC	—	—
mortality_16AM04	CC	AA	TT	GG	GG	GG	CC	CC	—
mortality_16AM05	CC	AA	—	GG	GG	GG	CC	—	TT
mortality_16CM01	—	—	—	—	—	—	—	—	—
mortality_16CM03	CC	—	—	—	—	—	CC	—	—
mortality_16CM04	CC	AA	AA	GG	GG	GG	CC	CC	—
mortality_16CM05	CC	—	TT	GG	—	GG	CC	CC	TT
mortality_16CM06	CC	AA	TT	GG	GG	GG	CC	CC	TT
mortality_16CM07	—	AA	—	—	—	—	—	—	—
mortality_16CM08	CC	AA	TT	GG	GG	GG	CC	—	TT
mortality_16CM09	CC	AA	TT	GG	GG	GG	CC	CC	TT
mortality_16CM10	CC	AA	—	GG	GG	—	CC	CC	TT
mortality_16CM11	CC	AA	TT	GG	GG	—	—	CC	—
mortality_16CM12	—	AA	—	GG	GG	GG	CC	CC	TT
mortality_16DM01	CC	AA	TT	GG	GG	GG	CC	CC	TT
mortality_16DM02	CC	AA	TT	GG	—	GG	CC	—	TT
mortality_16FS01	—	AA	—	GG	GG	GG	CC	CC	—
mortality_16FS02	CC	AA	TT	GC	GG	—	CC	CC	TT
mortality_16FS03	CC	AA	TT	GG	GG	GG	CC	CC	TT
mortality_16FS04	CC	AA	TT	GG	GG	GG	CC	CC	—
mortality_16FS05	—	—	—	—	—	—	CC	—	TT
mortality_16RL01	CC	AA	TA	GG	—	—	CC	CC	TT
mortality_16RL03	CC	AA	TT	GG	GG	GG	CC	—	TT
survivor_16RL05	CC	AA	—	GC	GG	CC	GG	CC	TT
survivor_16RL06	—	—	—	—	—	—	—	—	—
survivor_16RL07	CC	AA	AA	GG	AA	—	GG	TT	AA
survivor_16RL08	CC	AG	AA	GC	GG	CC	CG	CT	TT
survivor_16RL09	—	—	TT	—	—	—	CC	—	—
survivor_16RL10	CC	AG	TA	GG	AA	CC	CC	CC	TA
survivor_17PR01	TT	AA	TA	GG	GG	GG	CC	—	TA
survivor_17PR03	TT	GG	TA	CC	—	GG	CC	CC	TA
survivor_17PR04	—	—	AA	CC	GG	CC	CC	CT	TT

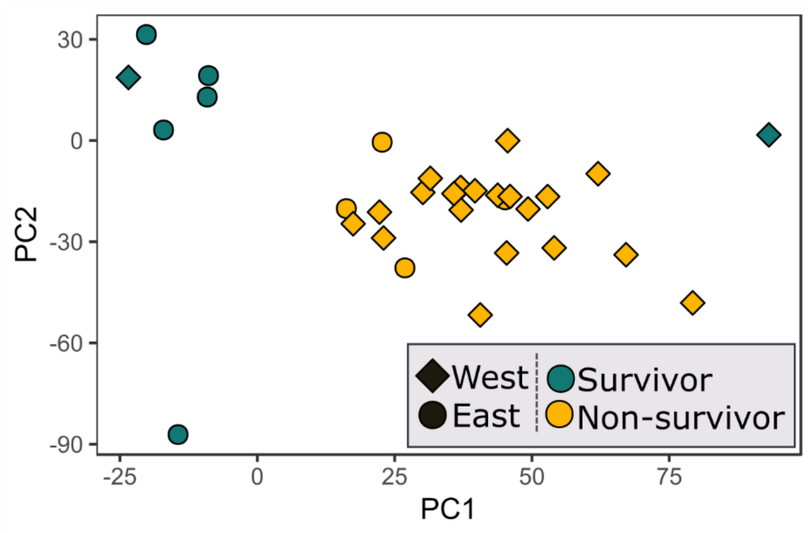

Fig. S1. A PCA performed in the same way as Fig. 2A, but with more stringent filters on missing data. Here, missing data is ≤ 8.7% per individual and ≤19% per locus (mean 1.9%), with a MAF limit 0.05. Applying these thresholds resulted in 13,666 loci and 31 individuals being included in the analysis. Survivors and non-survivors are shown in dark green and gold, respectively, with diamonds indicating individuals sampled from the western portion of the peninsula and circles indicating those from the east. PC1 explains 19% of the variance for survivors and 39% for the non-survivors, whereas PC2 explains 18% and 25%, respectively.

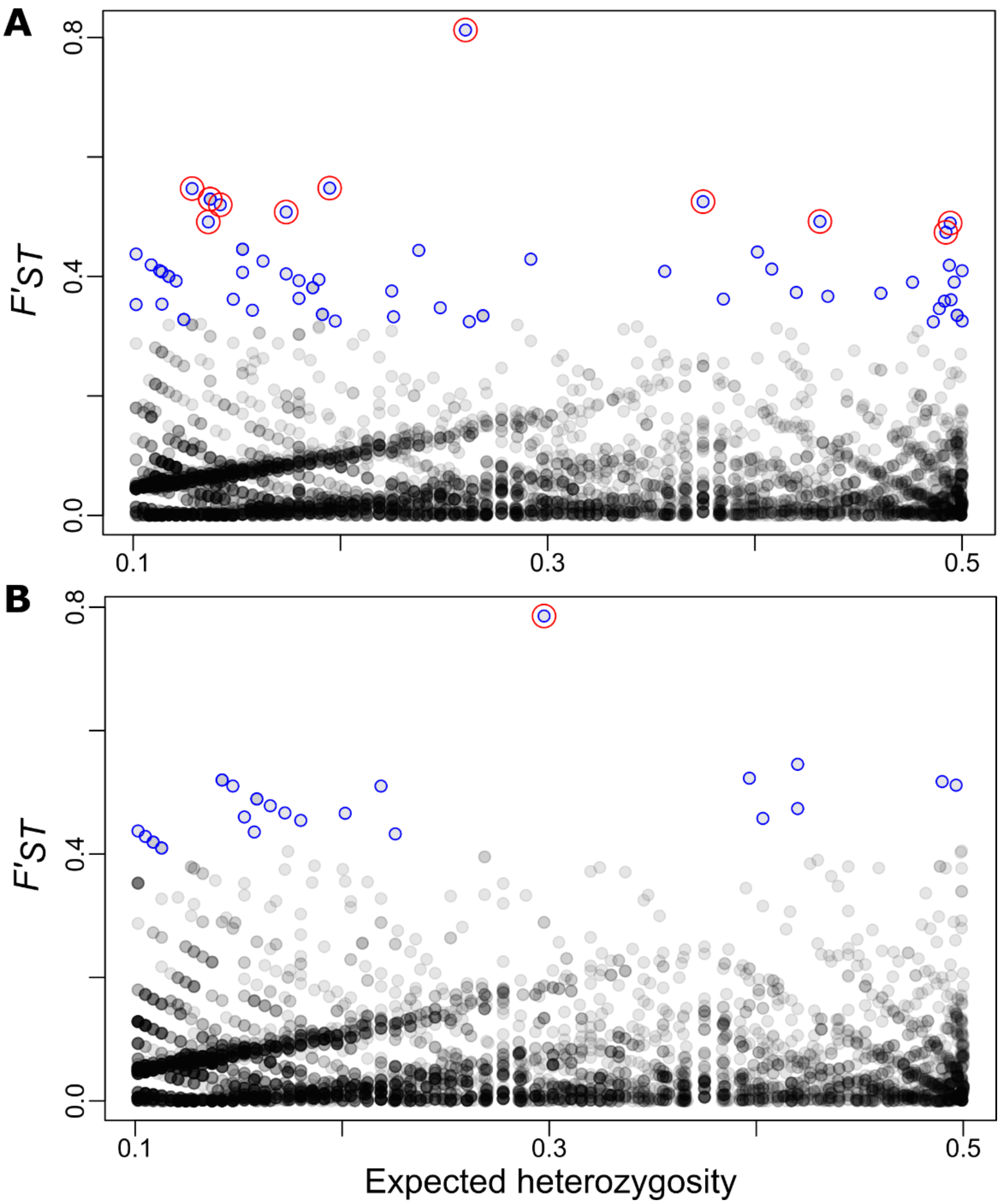

Fig. S2. Findings of OutFLANK, showing each SNP-site plotted in context of the expected heterozygosity (He) of the minor allele (x-axis) and the degree of differentiation between survivors and non-survivors (F'_{ST} on the y-axis, a version of F_{ST} not corrected for sample size as calculated in OutFLANK; Whitlock & Lotterhos, 2015) for analyses (A) of all individuals and (B) excluding individuals sampled in 2014. Loci in blue are significant at a threshold of alpha ≤ 0.05 (64 and 26 for A and B, respectively); loci with an additional red ring are significant at alpha ≤ 0.01 (12 and 1). Loci with low minor allele frequencies (He < 0.1) were excluded as per the developer's guidelines.

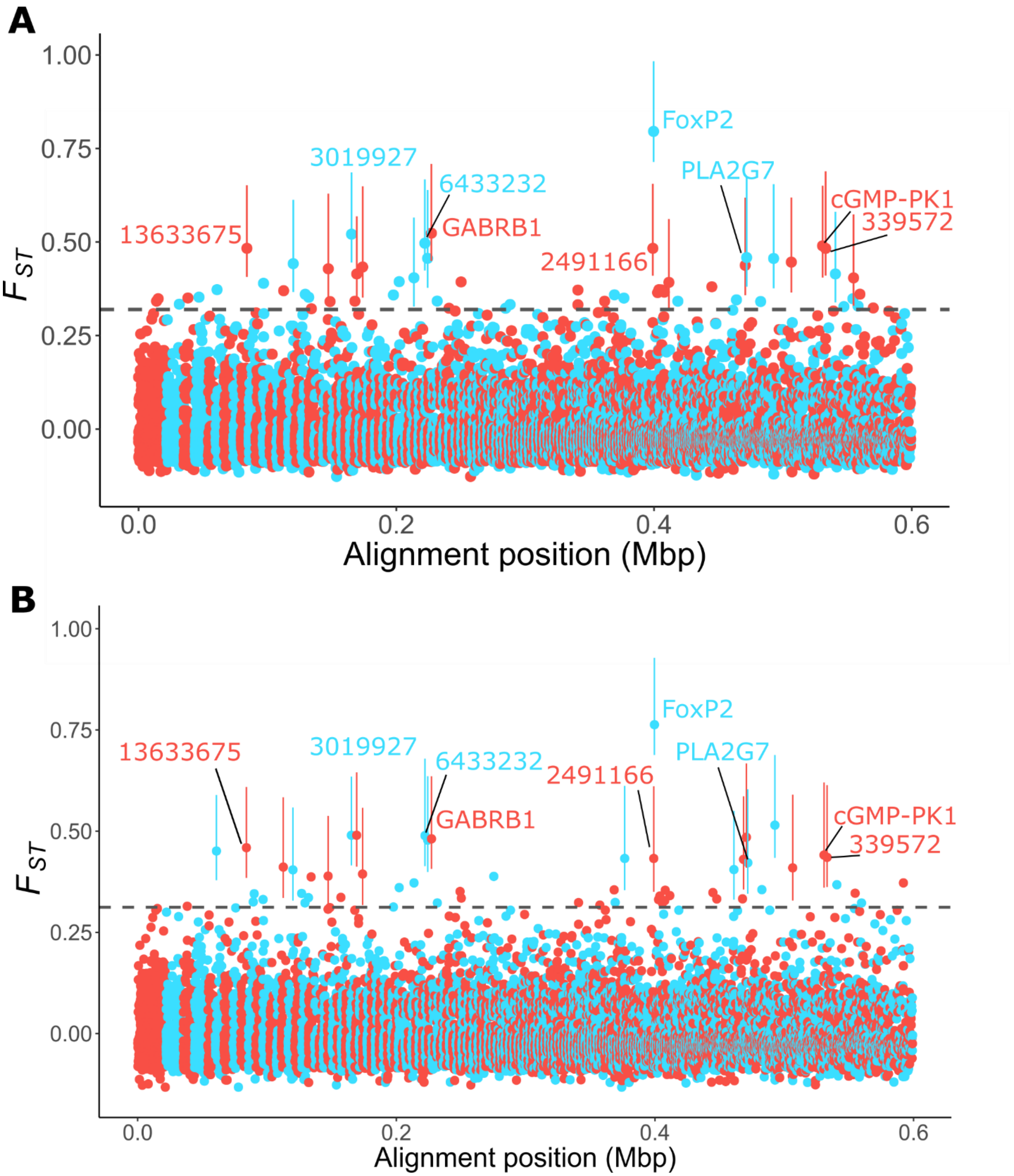

Fig. S3. F_{ST}-outlier plot with 95% confidence intervals based on analyses of (A) all the data, and (B) excluding the four bats from 2014 (see methods for details). The 9 top-candidate sites that were also identified in the other two tests (which are what we center the discussion upon) are labeled according to the gene name or position (see Table S2). In addition to these loci, 12 sites with CIs at least 5 standard-deviations from the mean were identified as significant in A (i.e., a total of 21 sites), and 13 additional sites were significant in B. However, these additional outliers are not discussed further due to their lack of identification via the two other outlier detection methods. Some outliers were moved horizontally for clarity.

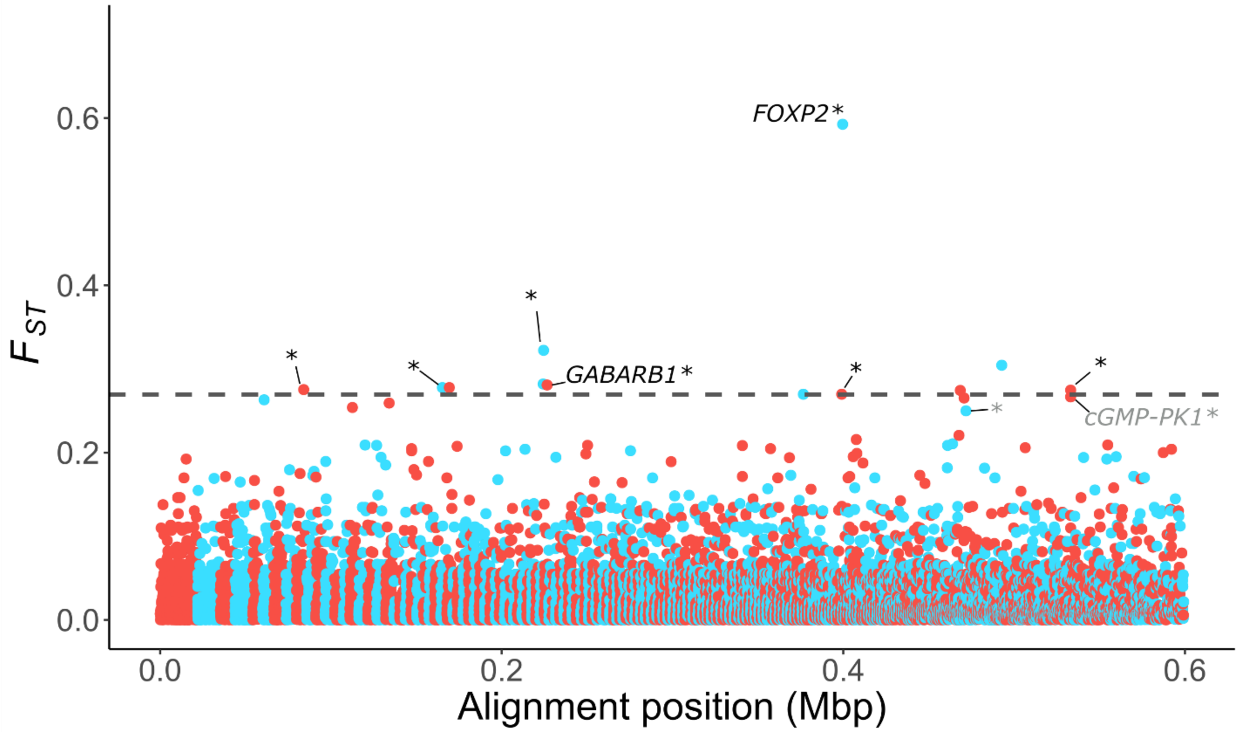

Fig. S4. Results of the F_{ST}-outlier analysis (see also Fig. 3) excluding the four bats from 2014 (see methods for details). Here twelve (as opposed to nine with all the data) significant outlier SNPs were detected, and two SNPs previously identified as significant based on nine-standard deviation are significant by five-standard deviations given the slight drops in F_{ST} (i.e., PLA2G7 and cGMP-PK1).

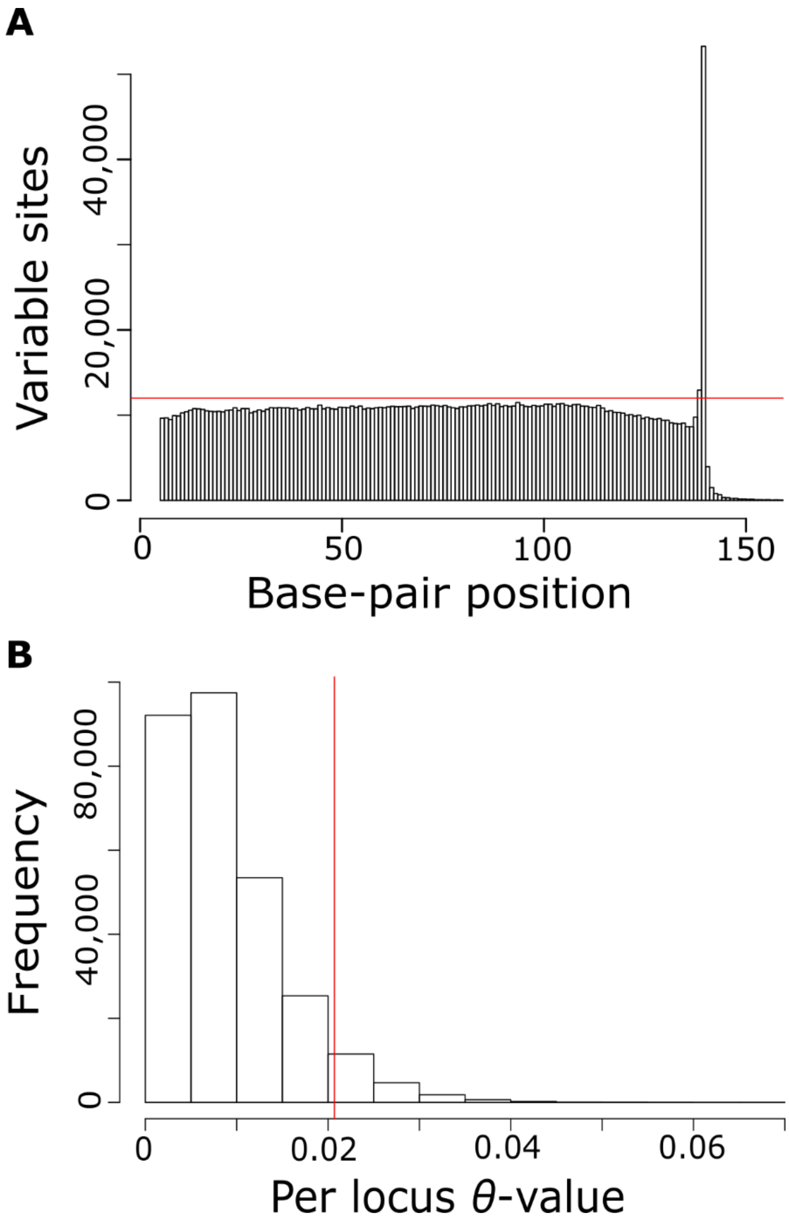

Fig. S5. A custom script in R was used to examine the number of variable sites per base-position across all reads (the ends of reads are more susceptible to sequencing errors). The pre-trimmed plot (A) indicates the threshold used for discarding SNPs (horizontal, at 4,500 variable sites) and (B) shows a subsequent trimming step in which the distribution of per locus θ-values was considered. Loci with θ-values above the 95% threshold (shown by the red line) were excluded in order to reduce the probability of including sites that were variable due to errors in sequencing

or assembly.

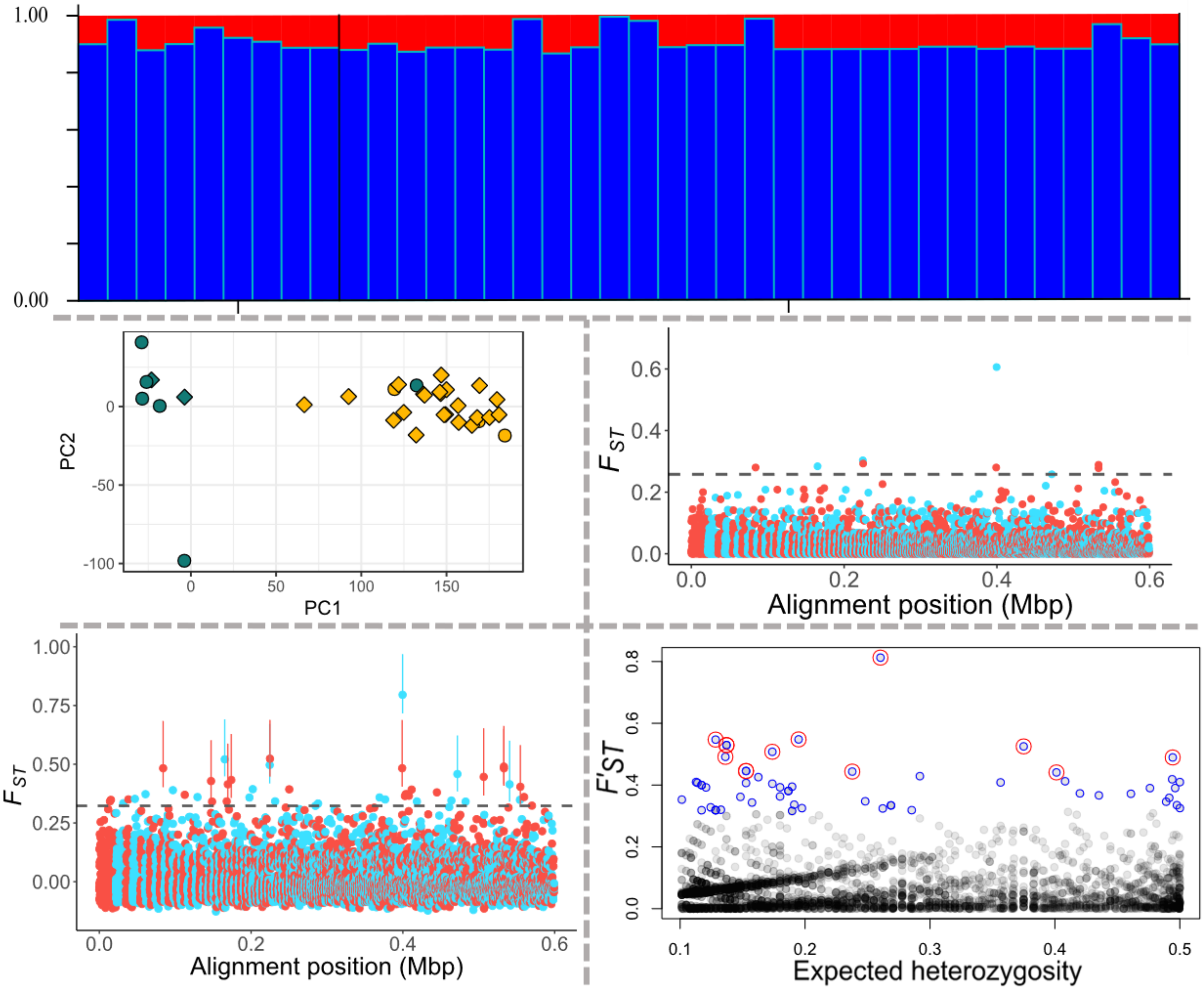

Fig. S6. The robustness of the results were confirmed by repeating analyses with a single randomly selected SNP per locus, except for the previously identified nine SNPs of interest. Evaluation of population subdivision in STRUCTURE[37] (top) shows a single, panmictic population (samples are in order corresponding to Table S1 with survivors on the left and non-survivors on the right). The PCA (middle left) corresponds to Fig. 2 with PC1 explaining 23% of the variance for survivors and 62% for non-survivors, and PC2 explained 13% for survivors and 9% for non-survivors. The estimated degree of drift using the *F*-statistic in STRUCTURE[36,37] (not pictured) remained similar at F=0.049 (SD±0.000624) for survivors and 0.0097 (±0.0007) for non-survivors. The outlier analysis (middle right; corresponding to Fig. 3) based on nine standard deviations from the mean (0.28) identified the same nine SNPs as significant, which were also significant in the bootstrap analysis (bottom left; see Fig. S3) and by OutFLANK[90] (bottom right; see Fig. S2).

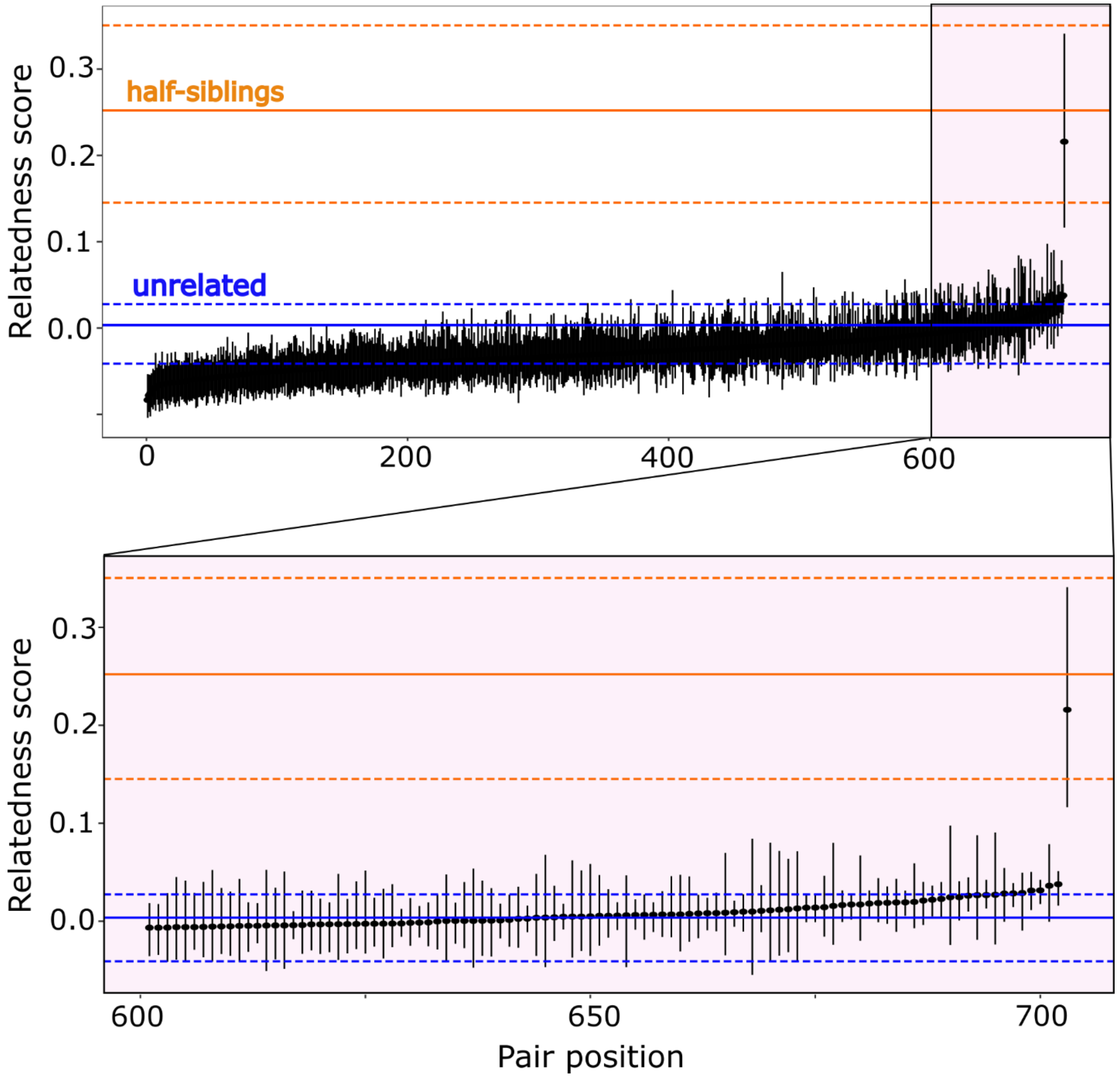

Fig. S7. Estimated relatedness of pairs of sampled bats (dots) with 95% confidence intervals (CIs, vertical lines), arranged along the x-axis from least to most related. The y-axis is Ritland's estimator of relatedness[77], which theoretically ranges from 0 to 1, with one representing a clone. Horizontal lines indicating expected scores for unrelated individuals (blue) and half-siblings (orange). Solid horizontal lines represent the mean expected value, whereas the dashed lines represent the 1st and 3rd quantiles. Estimates of expected and observed relatedness were generated using the package *related*[76]. Expected values were simulated from 250 randomly chosen loci from our dataset for 100 pairs of individuals per relationship category (parent-offspring, full sibling, half sibling, and unrelated), then estimated for each pair of samples based on all 1,242 loci used in the analyses (i.e., loci with a minor allele frequency greater than 0.01, and not missing from more than 2 individuals, with a minimum of 25% missing per individual). Only a single pair of individuals (sample IDs 16CM03 and 16CM11; see Table S1) was estimated to be related (as half-siblings; Ritland score = 0.216, 95% CI 0.12 – 0.34).

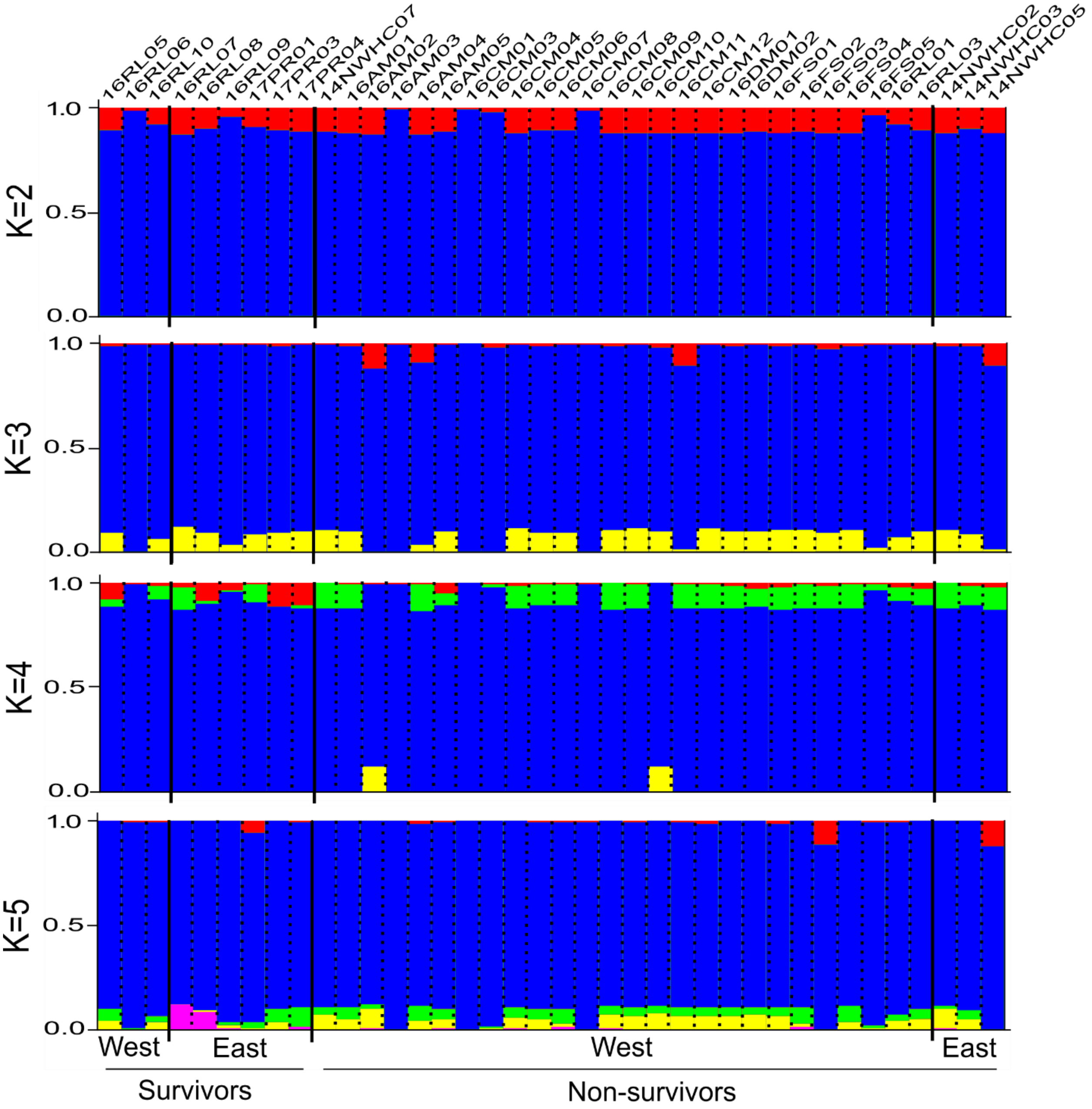

Fig. S8. A single genetic group is suggested by STRUCTURE[37], with no evidence of multiple ancestral source populations for different geographic areas. Results for the different *k*-genetic clusters are shown, where each individual (separated by dashed black lines) is represented by a bar and inferred ancestry (posterior probabilities of different ancestral makeups) is represented by different colors. Individuals are grouped by survivors (left) or non-surviving bats (right) and the geographic sampling region (West or East) are labeled. Sample labels (top) correspond to Table S1.

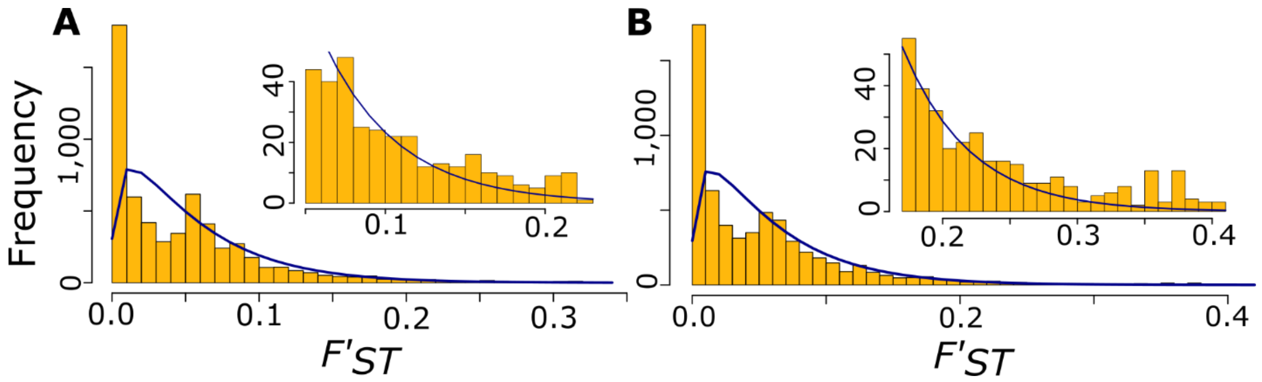

Fig. S9. Evaluations of the fit of the chi-squared distribution to the distribution of F'_{ST} values for purportedly neutral alleles in our dataset in OutFLANK[90] for (A) all individuals and ((B) excluding individuals sampled in 2014. Insets show a focus on the fit on the right tail of each distribution, which is the main concern (Whitlock & Lotterhos, 2015)—F'_{ST} values beyond this are considered outliers.
